# Molecular and agro-morphological characterization of ancient wheat landraces of turkey

**DOI:** 10.1186/s12870-017-1133-0

**Published:** 2017-11-14

**Authors:** Kahraman Gurcan, Fatih Demirel, Mehmet Tekin, Serap Demirel, Taner Akar

**Affiliations:** 1 Department of Agricultural Biotechnology, Faculty of Agriculture, Erciyes University, Kayseri, Turkey; 2 Department of Field Crops, Faculty of Agriculture, Igdır University, Igdır, Turkey; 3 Department of Field Crops, Faculty of Agriculture, Akdeniz University, Antalya, Turkey; 4 Department of Molecular Biology and Genetics, Faculty of Science, Yuzuncu Yil University, Van, Turkey

**Keywords:** Hulled wheats, Emmer wheat, Einkorn, Tir wheat, Characterization, Genetic diversity

## Abstract

**Background:**

Turkey is one of the important gene centers for many crop species. In this research, some ancient wheats such as tetraploid and diploid hulled wheats together with hexaploid tir wheats (*Triticum aestivum ssp. leucospermum* Korn.) landraces mainly adapted to harsh winter conditions of Eastern Anatolian region of Turkey were characterized at agro-morphological and molecular level. Totally 50 hulled wheat population from Kastamonu, Konya and Kayseri provinces and 15 tir wheats from Kars provinces of Turkey were in-situ collected for characterization in 2013. Some quantitative and qualitative traits of each population were determined.

**Results:**

Twenty three hulled wheat population collected from Kastamonu province were distinguished into nine emmer and 14 einkorn wheats at morphological level. Additionally, Konya, Kayseri and Kars population were characterized as einkorn, emmer and tir wheat, respectively. Among the evaluated traits, protein ratios of hulled wheats were strikingly higher than registered cultivars. All the populations were also examined by molecular level by using fluorescently labelled 11 polymorphic SSRs primers. The primers exhibited 104 bands, ranging from 6 to 16 with a mean value 9.45 per loci. The clustering analysis separated the germplasm into two clusters which were also divided into two subclusters based on genetic similarity coefficient. Sixty-five population and five checks were analyzed to estimate mean number of alleles (N), expected and observed heterozygoties (He and Ho), polymorphism information content (PIC), Wright fix index (F), genetic deviation from Hardy-Weinberg expectation (*Fit*-*Fis*) and genetic variation (*Fst*) were determined as 9.45, 0.71, 0.07, 0.67, 0.90, 0.39, 0.87 and 0.39, respectively. A clear genetic deviation from Hardy – Weinberg expectation was observed among population in particular. These results showed considerable genetic variation among landraces rather than within population.

**Conclusions:**

These molecular information has revealed genetically diverse einkorn, emmer wheat and tir wheat population could be used as parents for further breeding studies in both Turkey and abroad. Furthermore, the molecular analysis has also generally discriminated the germplasm into ploidy level.

## Background

Hulled wheats are the first domesticated crop species. Two hulled wheat species such as einkorn (*T. monoccocum* var. *monoccocum*) and emmer wheat (*T. dicoccum* L.) grains were firstly found in Cayonu excavation dated back to 6500–7000 B.C. in Turkey [1]. This clearly demonstrates that Turkey has a great experience on cultivation of these two hulled wheat species. They are also species in the bridge between cultivated (bread and durum) and wild wheats. Their spikes are not fragile, but they are hulled. Nowadays, mix of einkorn and emmer wheat population is mainly cultivated in rural areas of several provinces in Turkey such as Çankırı, Kars, Kastamonu, Kayseri, and Sinop. Tir wheats (*Triticum aestivum ssp. leucospermum* Korn.) are also unique bread wheat landraces especially for cultivation in harsh winter conditions of the Lake Van Basin including Kars province for centuries [2]. Due to their population habit, they have large variation for many agronomic traits and even enough resistance to some leaf diseases [3, 4]. Farmers living in remote areas of The Lake Van Basin especially in Kars province cultivate mix of hulled wheat and tir wheat, thereby it is commonly called as hulled wheat by local farmers and they are consumed as both food for humans and feed sources for animals, too.

Cultivation of hulled wheats had significantly decreased in the world as well as in Turkey due to their low yield level until 1960, but after 1990s, it has increased with regard to interest in natural and organic products in addition to health characteristics of hulled wheats associated with high starch-resistant content [5, 6]. Moreover, increasing awareness and demands for diversity and quality of food, hulled wheat species have become more important for organic farmers [7]. In addition to these, production of a wide range of regional products with higher nutritional value and suitability for processing resulted in different studies [8].

Effective use of plant genetic resources belongs to identification of phenotyphic and genotyphic variation of targeted germplasm. Determination of genetic diversity may result in effective use the germplasm especially for plant breeding studies. Diversity studies based on molecular markers are independent of environmental factor and can be estimated by using small amount of DNA [9]. Molecular diversity studies evaluate all levels of genetic structure from relationships among species complex components to the origin of particular genotypes [10]. For these purposes, different marker systems such as ISSR, AFLP, SSR and RAPD have been used for characterization of different cereal species including wheat germplasm. Simple sequence repeats (SSRs, also named as microsatellites) are polymorphic markers that are short and randomly repeated DNA sequences mostly founded in eukaryotic genomes [11]. There are a few studies on Turkish hulled wheats such as einkorn and emmer wheat and tir wheats at morphological [3, 4, 12, 13] and molecular level [14]. In this study, we examined the agro-morphological and molecular characteristics of ancient wheat species of einkorn, emmer and tir wheat population collected from on-farm conserved provinces of Turkey including commonly cultivated durum and bread wheat cultivars to uncover genetic diversity and relationship among population to be used as parents for further breeding programs.

## Methods

### Plant material

Totally, 50 hulled wheat populations from Kastamonu (23), Konya (16) and Kayseri (11) and 50 tir wheat population from Kars province of Turkey were in-situ collected. Additionaly, four durum (*T. durum* L.) cultivars, (Saricanak, C-1252, Y. population, and Kiziltan) and one bread wheat (*T. aestivum* L.) cultivar, Dogankent were included as controls in both field and laboratory experiments.

### Field experiment, observations and data analysis

Totaly 70 wheat genotypes were planted on the second of April of 2013 season at the Research Farm of Erciyes University, Kayseri, Turkey. Each entry was planted as two row, two- meter- plots with 35 cm row spacing [15]. Ten grams of the seeds from each entry were spring planted at 5 cm depth by hand and common agronomic practices were applied for cultivation of the wheat germplasm under rainfed conditions. Average temperature was lowest in April (13.0 °C) and highest in July (23.4 °C) and total seasonal precipitation was 90.8 mm. Average temperature value in the growing season was higher 3 °C than long term conditions and there was less 70.2 mm rain. In order to reduce the negative effect of severe drought during the cultivation period, the germplasm was routinly irrigated. The soil texture of the experimental area has argillaceous-sandy, slightly alkaline reaction and unsalted. The quantitative such as plant height, grain per spike, plant yield, heading time, maturity time, 1000 kernel weight and protein ratio and qualitative traits such as hairiness, waxiness, growth habitus and grain hull were measured in each population by taking five random single plant [16]. Basic statistical parameters, mean and standart deviation were computed for each entry in Minitab statistical software.

### DNA extraction and SSR amplification

Young leaves were harvested from all genotypes and used for DNA isolation using the CTAB method modified by [17]. In order to identify polymorphic SSR loci in ancient wheats, DNA of 12 hulled wheat population were amplified with 24 SSR primers [18–20]. PCR technique was performed in 15 μl volume containing: 7.8 μl dH2O, 1 μl primer (0.6 mM), 1.5 μl 10X PCR buffer (750 mM Tris-HCI pH 8.8, 200 mM (NH4)2SO4, 1.5 μl MgCl2 (25 mM), 1.5 μl dNTP, 0.2 μl Taq DNA polymerase (5 U/μl) ve 1.5 μl DNA (about 20 ng/μl). The amplification for initial screening was carried out in a thermocycler programmed as follows: 1 cycle of 95 °C/4 min denaturation, 35 cycles of amplification (94 °C/1 min, 58 °C/2 min and 72 °C/2 min), and 1 cycles of 72 °C/7 min final extention. The products were separated on 2% agarose gels, stained with ethidium bromide, photographed under ultraviolet light, and polymorphism noted visually. For the loci that appeared polymorphism in the initial screening, forward primers of 11 pairs, “Xgwm135, Xgwm312, Xgwm251, Xgwm149, Xgwm372, Xgwm493, WMC216, Xbarc180 [18], WMC170, WMC177 [19] and DuPw167 [20]” were fluorescently labeled with FAM, PED, VIC and NED and were used to amplify 70 genotypes using same PCR conditions as in the initial screening. For multiplexing, 1 μl of labeled products of each of four primers was combined with distilled water to a final volume of 200 μl. A 1 μl aliquot obtained from the 4 primer was loading ABI 3500 capillary electrophoresis instrument for fragment analysis.

### Marker scoring and data analyses

Allele sizes for 11 polymorphic SSR primer pairs were used to determine molecular characterization and determine genetic diversity among the 70 wheat population. For marker characterization, polymorphism information content (PIC), and fixation index (F = 1-Ho/He) were calculated for each microsatellite locus. Morever, *Fit*, *Fis* and *Fst* of Wright’s F-statistics for 11 SSR markers were calculated [21, 22]. *Fit* and *Fis* were described as genetic deviation from Hardy–Weinberg expectation within and among landraces, respectively. When *Fit* and *Fis* are 0, landraces are at Hardy–Weinberg equilibrium. *Fst*, ranging from 0 to 1, is an estimate of gene differentiation among landraces [23]. *Fst* is 0, if there is no genetic variation among landraces.

### Population structure and diversity analysis

Diversity analysis among wheat accesions was conducted using molecular data. To examine genetic relationships, the unweighted pair-group method with arithmetic mean (UPGMA) was performed to construct cluster based on maximum composite likelihood distance by Mega Software (7.0.14 version) [24]. We also conducted a principal coordinate analysis (PCoA) as an alternative approach to depict the genetic diversity among the accessions. PCoA of genetic data was performed using the PAST software (version 3.14). On the other hand, the population structure of the ancient wheat populations was conducted using the Bayesian approach and model-based clustering was generated by STRUCTURE software. Genotypic data were uploaded to STRUCTURE software and simulation parameters were determined. In order to detect the optimal value of K, consecutive Ks from 1 to 10 were run. For each K, 10 independent runs were carried out. The data were obtained from a 100.000 Markov Chain Monte Carlo (MCMC) replications after discarding initial 10.000 replications as burn-in replications [25] Structure Harvester (version 0.6.93) [26] was used to exhibit the optimal number of clusters “K”. Structure Harvester permits visualization of the STRUCTURE output to infer the number of clusters based on method described by [27] as well as Evanno’s method in the user manual [25].

## Results

### Phenotypic identification and variation

All germplasm were examined at morphologic level considering plant, spike and grain data collected during and after harvest in order to realize true identification. All wheat populations collected from Kars province had naked grain and they were identified as tir wheat while populations collected from Konya and Kayseri provinces had hulled grain “einkorn are emmer wheat”, respectively. However, 23 hulled wheat population collected from Kastamonu province were distinguished into nine emmer wheat and 14 einkorn. Mean plant height, number of grains per spike, plant yield, heading and maturity time, 1000 kernel weight and protein ratio of Kastamonu, Konya, Kayseri and Kars population were shown at Table 1. Additionally, qualitative traits such as hairiness, waxiness, growth habitus and grain hull were given at Table 2. Check cultivars had shorter plant height and maturity time but higher plant yield compared to all ancient wheat landraces from different ploidy levels due to long-term breeding studies (Table 1). However, diploid and tetraploid hulled wheats such as einkorn mainly from Konya and Kastamonu, emmer wheat from Kayseri and Kastamonu and tir wheat had longer plant height and maturity time but lower plant yield compared to check cultivars (Table 1). In terms of protein ratio, einkorn from Kastamonu (17.12%) and Konya (17.50%) and emmer wheat from Kastamonu (18.20%) and Kayseri (18.40%) in particular had incredibly high amount of protein when compared to check cultivars (11.08%) (Table 1). Emmer wheats collected from Kayseri (42.81 g) and Kastamonu (41.60 g) outyielded all wheats for 1000 kernels weight (Table 1). There was a clear relationship among plant yield and ploidy level that the more plant yield was obtained when ploidy level increased (Table 1).

**Table 1:** The mean and standard deviation of quantitative traits of ancient wheats

	Einkorn		Emmer		Tir wheat	Check cultivars	Mean
	Kastamonu	Konya	Kastamonu	Kayseri	Kars		
Plant height (cm)	71.3 ± 0.68	74.9 ± 1.22	72.3 ± 1.95	75.7 ± 1.81	82.2 ± 0.89	68.1 ± 0.53	74.08 ± 0.76
Number of grains per spike	25.6 ± 0.85	28.5 ± 0.89	17.5 ± 1.02	24.9 ± 2.18	26.9 ± 0.67	23.3 ± 1.53	24.45 ± 0.68
Plant yield (g)	0.57 ± 0.07	0.56 ± 0.08	0.74 ± 0.08	0.79 ± 0.09	0.94 ± 0.09	1.48 ± 0.11	0.86 ± 0.05
Heading time	76.10 ± 0.46	77.0 ± 0.45	69.20 ± 0.36	69.80 ± 0.57	67.20 ± 0.99	74.0 ± 0.82	72.22 ± 0.54
Maturity time	107.40 ± 0.37	110.50 ± 0.31	107.0 ± 0.29	107.30 ± 0.30	103.10 ± 0.35	94.60 ± 0.56	104.98 ± 0.68
1000 kernel weight (g)	36.0 ± 0.84	37.74 ± 0.32	41.60 ± 0.33	42.81 ± 0.32	34.80 ± 0.40	39.76 ± 0.54	36.79 ± 0.42
Protein ratio (%)	17.12 ± 0.14	17.50 ± 0.25	18.20 ± 0.31	18.40 ± 0.21	12.47 ± 0.20	11.08 ± 0.12	15.79 ± 0.39

**Table 2:** Some qualitative traits of ancient wheats

	Einkorn		Emmer		Tir wheat	Registered cultivars
	Kastamonu	Konya	Kastamonu	Kayseri	Kars	
Hairiness (%)	22.2	0	100	100	100	0
Waxiness (%)	44.4	0	100	100	100	0
Growth habitus	Prostrate/erect	prostrate	erect /prostrate	erect	erect	erect
Grain hull (%)	hulled	hulled	hulled	Hulled	naked	naked

In addition to quantitative variation, there was enough qualitative variation to distinguish ancient wheats from check cultivars. Einkorn from Kastamonu and Konya and emmer wheat from Kayseri and Kastamonu with grain hull, hairiness and waxiness were completely different from check cultivars in general (Table 2). However, einkorn samples from Kastamonu and Konya provinces were differentiated in terms of hairiness and waxiness, too (Table 2). Moreover, einkorn naturally has prostrate habitus while emmer wheat erect habitus but there were some mixed habitus especially from Kastamonu province in which farmers historically cultivate these two species altogether (Table 2).

### Measurement of genetic variation

Obtained data from 11 labelled SSR primers from different linkage groups (LG) were used for molecular characterization and estimation of basic population genetic parameters summarized at Table 3. Totally 104 alleles from 11 SSR loci were detected by screening 70 wheat population and check cultivars. The number of alleles per loci ranged from 6 to 16 with an average number of 9.45. The highest number of alleles was obtained for the loci WMC177 (NA), whereas Xgwm135 and Xgwm312 produced the lowest number of alleles (Table 3). The PIC value were used as an indicator of genetic diversity at each locus [28] changed between 0.50 and 0.86 with a mean of 0.67. The highest PIC value was produced by the locus WMC177, while the lowest of PIC value was produced by the locus Xgwm312. According to [29], the highest level of genetic diversity and the lowest level of genetic diversity were observed respectively in WMC177 (0.50) locus and Xgwm312 (0.98). The averege of genetic diversity was 0.67 based on PIC values. Wright’s fixation index (F) which estimates the inbreeding coefficient ranged from 0.69 for Xgwm135 to 1 for Xgwm372 with a mean value of 0.9. Landraces are accepted as heterozygous if only their F value reaches 1 [21, 22]. The *Fit*, *Fis* and *Fst* are also known as Wright statistical parameters were calculated to analyse the genetic structure by PopGene32 software. Mean of *Fis* was 0.87, varying from 0.32 (Xgwm135) to 1 (Xgwm372), and *Fit* was 0.39 on mean, ranging from 0.22 to 0.79 at corresponding loci and *Fst* was 0.39 on mean, varying from 0.23 (Xgwm251, Xbarc180, Xgwm251 and Xgwm493) to 0.80 (Xgwm312) (Table 3). The highest genetic variation among population was revealed by Xgwm312 with 0.80.

**Table 3:** The genetic variation of the entire landraces via SSR loci

Marker	LG	N	He	Ho	PIC	F	*Fis*	*Fit*	*Fst*
Xgwm135	1A	6	0.64	0.2	0.57	0.69	0.32	0.49	0.26
Xgwm312	2A	6	0.59	0.01	0.50	0.98	0.81	0.96	0.80
WMC170	2A	8	0.78	0.01	0.75	0.98	0.96	0.98	0.47
Xgwm251	4B	10	0.67	0.06	0.62	0.92	0.84	0.88	0.23
WMC177	2A	16	0.87	0.07	0.86	0.92	0.82	0.87	0.30
Xgwm149	4B	8	0.73	0.14	0.69	0.81	0.40	0.74	0.57
Xgwm372	2A	9	0.68	0,0	0.62	1.00	1.00	1.00	0.53
Dupw167	6A	8	0.73	0.06	0.70	0.92	0.86	0.90	0.33
Xgwm493	3B	10	0.61	0.03	0.56	0.95	0.87	0.94	0.23
WMC216	1B, 1D	13	0.85	0.06	0.83	0.93	0.67	0.75	0.25
Xbarc180	5A, 3B, 7A	10	0.69	0.14	0.67	0.8	0.91	0.93	0.23
Mean	–	9.45	0.71	0.07	0.67	0.9	0.87	0.39	0.39

### Genetic relationships among wheat accesions

**Fig. 1:**
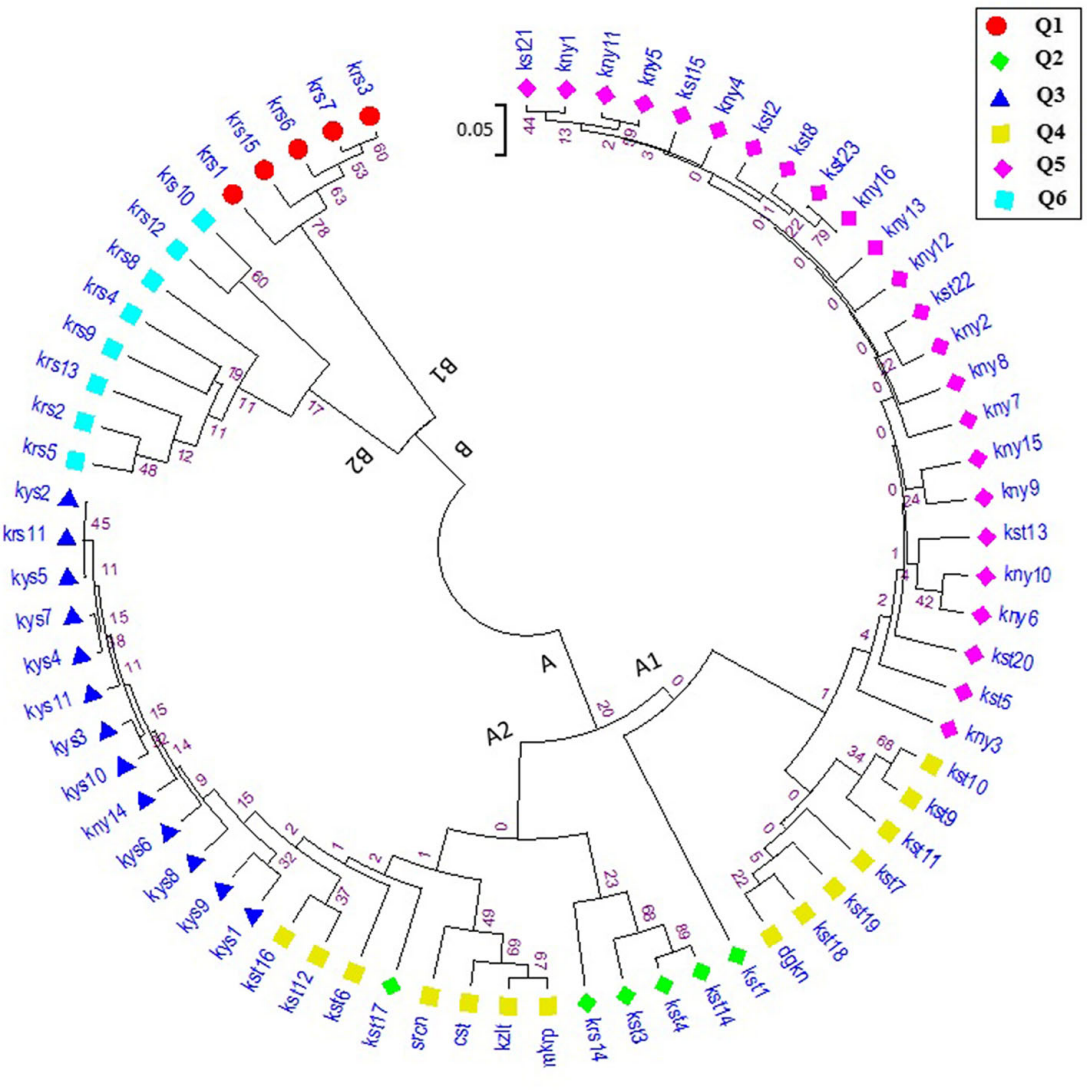
Dendrogram of 70 wheat accessions by UPGMA cluster analysis based on Bootstrap method

Obtained genetic distance [29] matrix was used to construct the dendrogram (Fig. 1). The 70 accessions were divided into main two groups, and these groups are named as A group and B group, respectively. Both groups were seperated into two sub-groups as well. The group A consists of mostly diploid *T. monococcum* and tetraploid *T. dicoccum* population and *T. durum* check cultivars while group B fully accounts for hexaploid tir wheats (Fig. 1). However, hexaploid wheat cultivar, Dogankent, was mis assigned into sub-group A1 together with tetraploid emmer populations collected from Kastamonu (Fig. 1). The highest maximum similarity was observed among emmer wheat (kayseri2-kayseri5-kayseri11) and einkorn (kastamonu23-konya16) accessions, respectively. The populations the most distinct from each other were kayseri2-kayseri7 and kastamonu 14-kastamonu4 in A2 group and kastamonu1 and kastamonu21-konya1 in A1 group (Fig. 1). Furtheremore, the B group was consisted of only hexaploid samples (tir wheats) obtained from Kars provinces. Tir wheats in this main cluster were also divided into two sub-clusters based on molecular data but they were not able to be differentiated at morphologic level. In addition to this, SSR data successfully distinguished mixed diploid (einkorn) and tetrapoid (emmer wheat) samples from Kastamonu provinces by only one exception (kastamunu 11) (Fig. 1). Interestingly, all tetraplois check cultivars consisted of a sub-sub cluster under subcluster A2.

### Population genetic structure and PCoA analysis

**Fig. 2:**
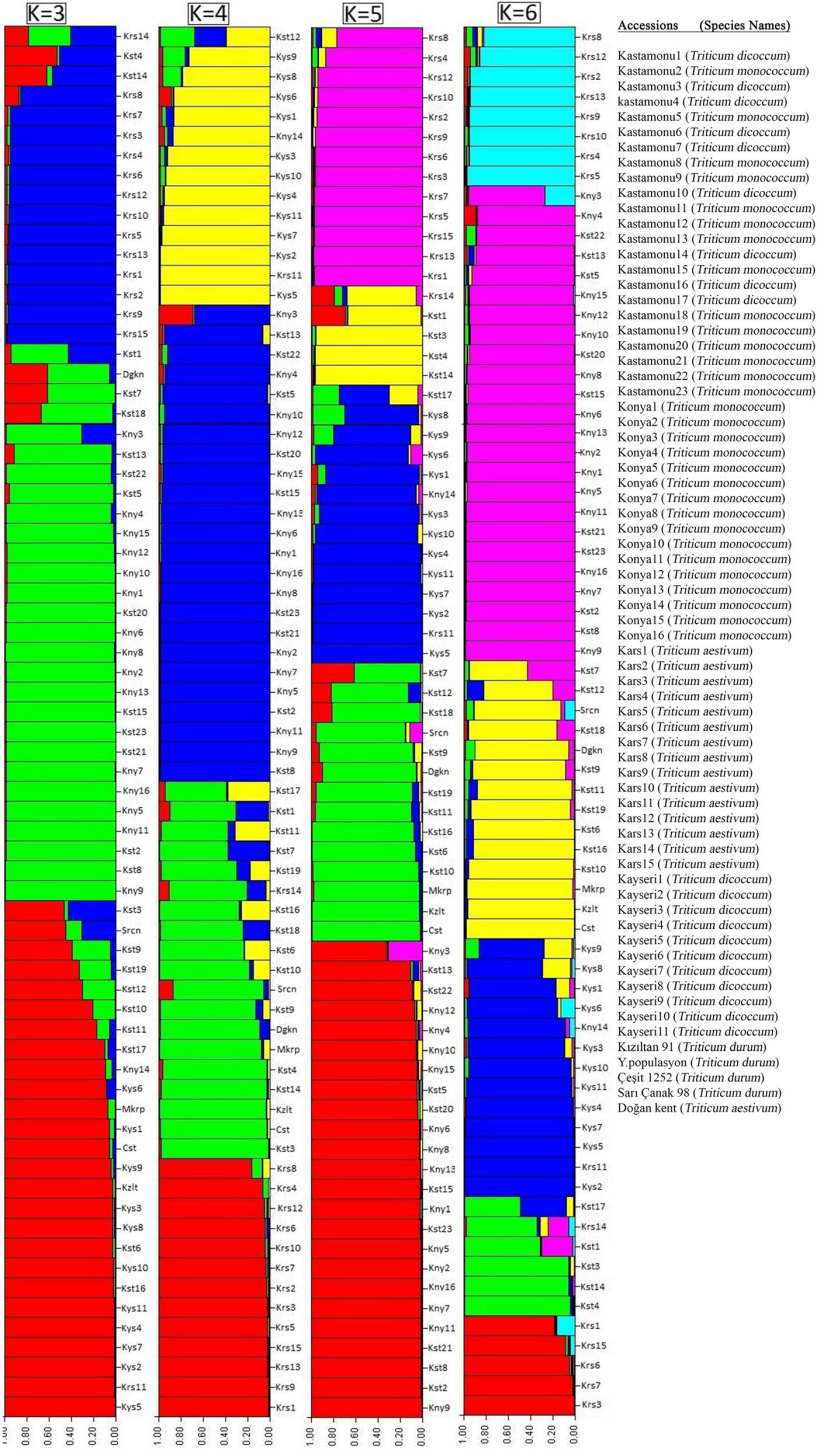
Genetic diversity structure of the 70 wheat accessions using the model-based Bayesian algorithm

In this study, population genetic structure was also examined among 65 ancient wheats and five registered varieties. This analysis was performed using all the 70 accessions and 10 independent runs of STRUCTURE for each K value (hypothetical number of subpopulations) from 1 to 10. For each K value, the run showing the highest posterior probability of data was considered as STRUCTURE output data. Then, these data were loaded to Structure Harvester and the results suggested that optimal number of K = 6 implying the existence of six main groups among the wheat gene pool. On the other hand, clustering pattern is visualized in a graph to demonstrate the population structure (Fig. 2). Analyses demonstrated that the populations in-situ collected from Turkey together with check cultivars were divided into six different subpopulations.

The structure program assumes that individuals with a membership coefficient of above 0.80 is regarded as pure and accessions with a membership coefficient of below 0.79 were considered as hybrids. A total of 55 accessions which is 0.80 and above of population membership coefficient was identified. Using a membership coefficient, 24 accesions were assigned into the largest subpop5 (Q5), with einkorn (*T. monoccocum*) accessions mostly from Kastamonu and Konya. Hexaploid tir wheat (*Triticum aestivum ssp. leucospermum*) accessions mostly clustered to Q1 and Q6 except for kars11 and kars14 together with registered hexaploid cultivar Dogankent which were situated in different subpopulations such as Q3 and Q4, respectively (Fig. 2). Subpopulation Q3 mostly comprised of accessions collected from Kayseri province and exceptly konya14 and kars11. The registered varieties were divided into subpopulation Q4 (Fig. 2). In order to measure distances among the subpopulations and to further evaluate population structure, net nucleotide distances among pairs of sub-populations were computed (Table 4).

**Table 4:** Allele-frequencies divergence among subpopulations based net nucleotide distances

Pop ID	Q1 population	Q2 population	Q3 population	Q4 population	Q5 population	Q6 population
Q1 population	******					
Q2 population	0.0853	******				
Q3 population	0.1238	0.0620	******			
Q4 population	0.0904	0.0303	0.0427	******		
Q5 population	0.1002	0.0535	0.1095	0.0486	******	
Q6 population	0.0771	0.0446	0.0892	0.0634	0.829	******

The distances between pairs of sub-populations ranged from 0.0303 (between Q2 and Q4) to 0.128 (between Q1 and Q3). When considering K = 4, accessions were mainly seperated four subpopulations according to regions that they were collected (Fig. 2). The separation of wheat accessions was likely realized based on their polyploid levels such as diploid, tetraploid and hexaploid, respectively (Fig. 2).

**Fig. 3:**
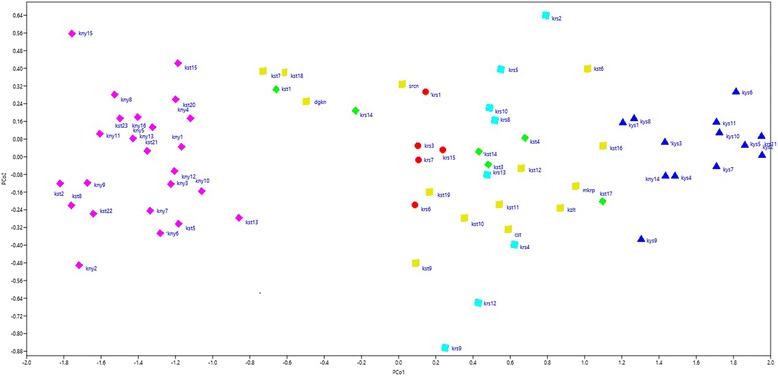
Principal coordinate analysis of the 70 wheat accessions based on 11 SSRs loci genotyping

As an alternative method to reveal genetic diversity, PCoA analysis also divided the 70 ancient wheat accession together with check cultivars into six major groups which were consistent with results of STRUCTURE (Fig. 3). The majority of populations collected from the same region were grouped more closely on the PCoA graph. The accessions belonging to Q5 were essentially distributed in the upside left part of the plot while the accessions from Q3 distributed in the right-side of plot. The accessions belong to Q2 and Q3 subpopulations showed dispersed distribution in the coordinate system. The five accessions of ancient wheat (konya5, kars9, kars2, kars12 and konya2) were located quite distant from the remaining accessions. The accessions from the landraces and registered varieties within Q4 were more distant each other indicating that higher genetic diversity remains in these landraces.

## Discussion

### Phenotypic variation

Cereal landraces and especially ancient wheats such as einkorn, emmer and tir wheat are unique germplasm for adapting unfavorable conditions and at the same time they are good sources for disease and pest resistance together with reasonable phytonutrients. There was an obvious difference in terms of plant height, maturity time and grain yield between the modern cultivars and the ancient wheats in our field experiment. The ancient wheats in particular and the landraces are characterized as longer plant height, later maturity and lower grain yield compared to modern check cultivars. Due to the fact that all modern checks had semi-dwarf genes, they have been adapted nutrient rich conditions resulting in high grain yield and optimum maturity time. Our observations were in fully compromise with [30–32]. Moreover, protein content of einkorn and emmer wheat was superior to that of modern cultivars. This can be explained that not only some agronomically important traits but also grain quality traits which have been decreased during term breeding of modern cultivars [32, 33]. Still, hulled wheats are extraordinary gene sources for many agronomical traits including high protein content identified in wild emmer wheat [34]. Modern cultivars have lost some morpho-physiological characters such as hairiness on leaves and waxiness on stems and leaves which can contribute drought tolerance of the genotypes during the selection process. We observed that nearly all hulled wheat and tir wheat population had hairy and waxy compared to modern wheat as mentioned by [32].

### Genotypic variation and population genetics parameters

Fragment analyses of the most polymorphic 11 SSR markers by using 70 wheat accessions produced a total of 104 alleles to be used for determination of genetic variation among and within populations. The allelic information data comprised of N, He, Ho, PIC, F and *Fst* were separately calculated for the all accessions (Table 3). PIC value which ensures an information in about discriminating power of a marker [35] ranged from 0.5 (Xgwm312) to 0.86 (WMC177), with a mean of 0.67. This mean PIC value is higher than those were reported for Indian emmer wheat accessions [36], Ethiopian emmer landraces [37] and Italian emmer wheat accessions [38], but lower than the value of 0.68 reported by [39] for 283 of wheat accessions. The allele number of per loci was ranged from 6 (Xgwm135 and Xgwm312) to 16 (WMC177).We observed that the average number of alleles per locus was 9. This mean value was lower than a mean of 18 allele number per locus in wild emmer wheat populations reported by [40]. This can be attributable to wild nature of wild emmer wheat collected from Israel. However, this was higher than the average allele number, 5.2 in the 30 spelt wheat accessions detected with 17 SSR markers by [41, 42] reported that a mean allele number per locus was 18.1 which was determined with 24 SSR markers in 998 accessions of *T. aestivum*. This highly level of heterozygosity in the last report comparing our result can be explained by highly diverse and large amount of bread wheat landraces accessions compiled worldwide.

Mean He value was 0.71 (0.59–0.87) for all wheat accessions in this experiment. According to previous studies, He value was 0.650 (0.211–0.899) for Eurasian bread wheat varieties [43]; 0.70 (0.46–0.82) for Siberian common spring wheat [44] and 0.56 (0.18–0.80) for Chinese wheat gene pool [45]. Considering characteristic features of the 11 SSR primer, He value for was lower the loci with low number of allele, whereas the He value was higher for the loci with high number of allele. These results are in consistent with results reported by [46].

Mean *Fst* value was 0.39 and changed from 0.23 to 0.80 in this study. The data explain that 39% of variation came from among population but 61% of variation from within population and this was higher than durum wheat landraces (0.25) and mustard landraces (0.12) reported by [31, 47], respectively. Mustard is an open pollinated crop species thus there was more variation inside the population compared to self-pollinated ancient wheats [22, 47]. Additionally, use of three different ploidy level of wheats compared to durum wheat landraces [31] resulted in more variation among ancient wheat population species in this study.

### Genetic relationship and population structure

Population structure is a component which is most convenient for analyzing genetic structure and a key step for further association studies [48]. Cluster analysis using a model-based method on SSR displayed that all accessions were classified into main two clusters which were divided into groups correspondence with result of STRUCTURE analysis (Fig. 1). Main cluster-A consisted of 57 mainly diploid and tetraploid wheat accessions that can be further clustered into four subgroups and main cluster-B comprised of 13 hexaploid tir wheat accessions that were further clustered into two subgroups. This showed effectiveness of SSRs to mainly discriminate crop species into their ploidy levels. Moreover, two subgroups in cluster B could be first clue of different subspecies among tir wheat accessions so further study is required to clarify this issue. The latest study on identification of subspecies of *Juniperus thurifera* L. by [49] is in agreement with this result. In this study, the population structure classified ancient wheat genotypes into six subpopulations using ΔK value of data obtained from STRUCTURE software according to [25]. The largest group included 24 einkorn accessions mainly originated from Kastamonu and Konya provinces. The smallest subpopulation consisted of 5 tir wheat accessions obtained from Kars. When considering a membership probability threshold of 0.60, 6 accessions were clustered by STRUCTURE to Q2, 13 to Q3, 14 to Q4 and 8 to Q6 (Fig. 2). Furthermore, Principal Coordinate Analysis exhibited that 70 accessions was separated into six major groups consistent with results composed by STRUCTURE (Fig. 3). Similar results obtained from UPGMA cluster and STRUCTURE analyses verified population structure and genetic diversity assessment. Distribution of the 70 ancient wheat accessions in PCoA was also supported by the POPULATION STRUCTURE and as well as the dendrogram. Moreover, it can be suggested from this study that population structure was also correlated with geographic origin of the ancient wheat germplasm. The same results was obtained by [50] who reported that 2 main geographic groups in wild emmer (*T. dicoccoides*) so they suggested that domestication of tetraploid hulled wheat most probably occurred in the Karacadag region of Turkey.

## Conclusion

The field experiment under drought conditions showed that einkorn and emmer wheat populations with longer plant height, more 1000 kernel weight and protein ratio together with hairy and waxy leaves and stem characteristics can be used as genitor to develop more drought tolerant new germplasm. Moreover, SSR markers were an effective tools to determine genotypic variation, genetic relationship, different ploidy level and genetic distance among ancient wheat populations. To reveal genetic divergence among different population, both population structure and principal coordinate analyses can be effectively used based on genotypic data. On the other hand, a clear genetic deviation from Hardy – Weinberg expectation among ancient wheat population was good indicator for selecting superior genotypes. We suggest that effectiveness of SSR markers for the separation of wheats in different ploidy level as was in this study be tested in further studies.

## Abbreviations

F: Wright fix index
*Fit*-*Fis*: *G*enetic deviation from Hardy-Weinberg expectation
*Fst*: *G*enetic variation
He: Expected heterozygoties
Ho: Observed heterozygoties
N: Number of alleles
PIC: Polymorphism information content
